# How relationship-oriented behavior influences employee voice-silence conversion in cross-cultural virtual teams: the mediating role of psychological safety

**DOI:** 10.3389/fpsyg.2025.1662897

**Published:** 2026-01-20

**Authors:** Xuetong Dong, Luling Xing

**Affiliations:** 1 School of Management, Qilu University of Technology, Jinan, China; 2 Department of Business Administration, The University of Suwon, Hwaseong-si, Republic of Korea

**Keywords:** cross-cultural virtual teams, digital workplace, leadership effectiveness, psychological safety, relationship-oriented behavior, voice-silence conversion

## Abstract

**Introduction:**

This study examines how leader relationship-oriented behavior influences employee voice-silence conversion in cross-cultural virtual teams through digital contexts.

**Methods:**

Using structural equation modeling with data from 342 team members across 68 virtual teams spanning 23 countries, we test a moderated mediation model that positions psychological safety as a key mediating mechanism.

**Results:**

Results demonstrate that relationship-oriented behavior significantly promotes voice-silence conversion toward increased voice behaviors (*β* = 0.34, *p* < 0.001), with psychological safety serving as a critical mediator (indirect effect *β* = 0.24, *p* < 0.001). Cultural value differences negatively moderate this relationship (*β* = −0.18, *p* < 0.05), while digital tool usage frequency enhances the effectiveness of relationship-oriented behaviors (*β* = 0.15, *p* < 0.05).

**Discussion:**

The findings extend leadership theory by demonstrating how traditional relationship-oriented approaches can effectively operate in digitally-mediated cross-cultural contexts, while advancing understanding of voice-silence as dynamic conversion processes rather than static behavioral states. Practical implications suggest that organizations should prioritize relationship-building competencies among virtual team leaders while recognizing that cultural diversity requires adaptive leadership approaches and that digital technologies can amplify rather than constrain interpersonal leadership effectiveness.

## 1 Introduction

The rapid advancement of digital technologies has fundamentally transformed organizational structures and work arrangements, leading to the widespread adoption of virtual teams as a dominant form of collaboration across industries (Abakpa and Dvouletý, 2025). In this digital era, organizations increasingly rely on cross-cultural virtual teams to leverage global talent pools, reduce operational costs, and enhance competitive advantages in international markets (Taras et al., 2021). However, the geographic dispersion and cultural diversity inherent in these teams present unique challenges for effective leadership and communication, particularly regarding employee voice and silence behaviors that are critical for organizational learning and innovation (Morrison, 2023).

Cross-cultural virtual teams operate within complex environments where cultural differences, technological mediation, and physical separation create barriers to effective interpersonal relationships and communication (Liu, 2025). However, existing voice and silence research has predominantly examined these phenomena in traditional face-to-face settings, leaving critical questions unanswered about how these dynamics operate in digitally-mediated cross-cultural contexts (Gilson et al., 2015). The fundamental problem is that conventional voice theories assume stable, co-located work environments with consistent cultural norms and rich face-to-face interactions—assumptions that do not hold in contemporary virtual teams where team members from diverse cultural backgrounds must navigate communication through digital platforms that filter and potentially distort interpersonal cues.

Leader relationship-oriented behavior, characterized by consideration, support, and interpersonal sensitivity, has emerged as a particularly crucial leadership approach in virtual team contexts (Lane et al., 2024). Yet current theories fail to adequately explain how such behaviors can effectively bridge cultural gaps and technological barriers simultaneously. The digital transformation introduces a theoretical paradox: while relationship-oriented leadership relies heavily on interpersonal warmth and emotional connection, virtual environments reduce the richness of interpersonal communication that traditionally enables these behaviors (Edmondson, 1999). Moreover, cultural diversity compounds this challenge, as the meaning and effectiveness of relationship-oriented behaviors vary significantly across different cultural contexts, potentially amplifying or diminishing their impact on employee communication in ways that existing theories do not fully address.

Most critically, current research treats employee voice and silence as separate, static behavioral choices rather than examining the dynamic conversion process between these states. This static perspective is particularly problematic in cross-cultural virtual contexts where employees continuously adjust their communication strategies based on rapidly shifting perceptions of psychological safety, leader support, and cultural appropriateness (Morrison, 2014). The existing literature cannot explain why some employees in virtual teams frequently shift between voice and silence while others maintain consistent patterns, nor can it clarify how relationship-oriented leadership influences these dynamic transitions when filtered through both cultural differences and technological mediation. This represents a significant theoretical gap that limits our understanding of communication effectiveness in one of the fastest-growing organizational forms.

Despite the growing importance of cross-cultural virtual teams in contemporary organizations, research examining the relationship between leader relationship-oriented behavior and employee voice-silence conversion remains limited. Previous studies have primarily focused on traditional face-to-face team settings or have examined voice and silence as separate phenomena rather than exploring their dynamic interplay. This research gap is particularly pronounced in the context of digitally-mediated cross-cultural environments, where unique challenges and opportunities exist for leadership influence on employee communication behaviors.

The primary research question guiding this study is: How does leader relationship-oriented behavior influence employee voice-silence conversion in cross-cultural virtual teams under digital contexts? Specifically, this research aims to examine the mechanisms through which relationship-oriented leadership behaviors facilitate or inhibit the dynamic transitions between employee voice and silence, while considering the moderating effects of cultural diversity and digital communication contexts.

This study makes several theoretical and practical contributions to the literature. Theoretically, it extends leadership theory by examining relationship-oriented behaviors in digitally-mediated cross-cultural contexts, while advancing our understanding of voice-silence dynamics as a conversion process rather than discrete behaviors. Practically, the findings will provide valuable insights for managers leading cross-cultural virtual teams, offering evidence-based strategies for fostering effective communication and leveraging diverse perspectives in digital work environments.

The remainder of this paper is organized as follows: Section II presents a comprehensive literature review and theoretical framework development; Section III outlines the research methodology and data collection procedures; Section IV presents the empirical findings and analysis; Section V discusses the theoretical implications and practical applications; and Section VI concludes with limitations and directions for future research.

### 1.1 Current research status of cross-cultural virtual team leadership theory

Cross-cultural virtual teams represent a distinctive organizational form characterized by geographically dispersed members from different cultural backgrounds who collaborate primarily through digital communication technologies to achieve shared objectives (Malhotra et al., 2007). These teams combine the complexities of cultural diversity with the challenges of virtual collaboration, creating unique dynamics that differentiate them from traditional co-located teams or mono-cultural virtual teams (Rosen et al., 2007). The defining characteristics of cross-cultural virtual teams include temporal and spatial dispersion, reliance on technology-mediated communication, cultural heterogeneity among members, and the absence of regular face-to-face interactions (Kirkman et al., 2002).

The theoretical foundation of virtual team leadership has evolved significantly over the past two decades, initially drawing from traditional leadership theories before developing more specialized frameworks (Avolio et al., 2014). Early research approached virtual team leadership through the lens of transformational and transactional leadership models, focusing on how established leadership behaviors could be adapted to technology-mediated environments (Bass and Avolio, 1990). However, scholars gradually recognized that virtual contexts required fundamentally different leadership approaches, leading to the development of virtual-specific leadership theories that emphasized communication frequency, trust-building, and relationship management (Cascio and Shurygailo, 2003).

The emergence of cross-cultural dimensions in virtual team research has further complicated the theoretical landscape, as cultural differences in communication styles, power distance, and collectivism–individualism orientations significantly influence leadership effectiveness (Hofstede, 1980). Contemporary virtual team leadership theory has thus evolved to incorporate cultural intelligence, cross-cultural communication competence, and culturally adaptive leadership behaviors as core components of effective virtual team management (Earley and Gibson, 2002).

Relationship-oriented behavior in virtual team contexts manifests through several distinct mechanisms that differ from traditional face-to-face leadership interactions. Virtual leaders must demonstrate consideration and support through technology-mediated channels, requiring heightened attention to communication clarity, response timeliness, and emotional expression through digital platforms (Maznevski and Chudoba, 2000). The absence of nonverbal cues in many virtual interactions necessitates that relationship-oriented leaders develop compensatory behaviors, such as increased communication frequency, explicit expression of empathy, and proactive check-ins with team members to maintain interpersonal connections (Martins et al., 2004).

In cross-cultural virtual environments, relationship-oriented behaviors must also account for cultural variations in communication preferences and relationship expectations. Leaders must adapt their supportive behaviors to align with different cultural norms regarding hierarchy, directness, and interpersonal relationships, while simultaneously fostering an inclusive team climate that accommodates diverse cultural perspectives (Neeley, 2015). The digital mediation of these relationships creates additional layers of complexity, as technology filters and potentially distorts the transmission of relationship-building behaviors across cultural boundaries.

Despite significant theoretical advances, existing research on cross-cultural virtual team leadership exhibits several notable limitations. First, most studies have focused on Western cultural contexts, with limited exploration of leadership dynamics in teams spanning multiple cultural regions or including members from non-Western cultures (Kayworth and Leidner, 2001). Second, the majority of research has examined leadership behaviors as static constructs rather than dynamic processes that evolve over time in response to changing team needs and contexts. Third, there has been insufficient attention to the bidirectional nature of leader-member interactions in virtual environments, particularly how cultural differences in follower behaviors influence leadership effectiveness.

Furthermore, current theoretical frameworks have not adequately addressed the temporal aspects of relationship development in cross-cultural virtual teams, where trust and rapport building may follow different trajectories compared to traditional team settings. The rapid pace of technological change has also outpaced theoretical development, creating gaps in understanding how emerging digital collaboration tools and platforms influence leadership processes and relationship dynamics.

Future research directions in cross-cultural virtual team leadership theory should focus on developing more culturally inclusive frameworks that account for diverse leadership and followership styles across different cultural contexts. Additionally, there is a need for longitudinal studies that examine the evolution of leadership relationships over time in virtual environments, as well as research that explores the specific mechanisms through which digital technologies facilitate or constrain relationship-oriented leadership behaviors in culturally diverse teams.

### 1.2 Research on employee voice-silence behavior conversion mechanisms

Employee voice behavior encompasses the voluntary expression of ideas, suggestions, concerns, or opinions about work-related issues with the intention of improving organizational functioning or challenging problematic practices (Van Dyne and LePine, 1998). This conceptualization has evolved to include multiple dimensions, with scholars distinguishing between promotive voice that focuses on constructive suggestions for improvement and prohibitive voice that challenges existing practices or raises concerns about potential problems (Liang et al., 2012). Additionally, voice behaviors can be categorized based on their targets, including upward voice directed toward supervisors, lateral voice shared with colleagues, and downward voice communicated to subordinates.

Employee silence, conversely, represents the intentional withholding of ideas, information, or concerns that could potentially benefit the organization or address important issues (Van Dyne et al., 2003). Silence behaviors are not merely the absence of voice but constitute active choices influenced by various psychological, social, and organizational factors (Pinder and Harlos, 2001). Research has identified distinct types of silence, including acquiescent silence characterized by passive resignation, defensive silence motivated by fear of negative consequences, and prosocial silence intended to protect others or the organization from potential harm.

The dynamic nature of voice-silence conversion is influenced by multiple temporal and situational factors that create fluctuating thresholds for employee communication behaviors (Milliken et al., 2003). Individual characteristics such as personality traits, cultural background, and past experiences interact with contextual variables including leadership style, organizational climate, and perceived psychological safety to determine the likelihood of voice versus silence in specific situations (Morrison and Milliken, 2000). Employees engage in a continuous cognitive evaluation process, weighing the potential benefits of speaking up—such as contributing to problem-solving, gaining recognition, or fulfilling professional obligations—against the perceived risks including social penalties, career consequences, or relationship damage.

Several key factors influence the voice-silence conversion mechanism, including psychological safety, which serves as a fundamental prerequisite for voice behavior by reducing employees’ fears of negative consequences (Edmondson and Lei, 2014). Leader-member exchange quality significantly affects this conversion process, as high-quality relationships tend to lower the perceived costs of voice while increasing the expected benefits of speaking up. Cultural factors also play a crucial role, with collectivistic cultures often exhibiting higher silence tendencies due to emphasis on harmony and face-saving, while individualistic cultures may encourage more direct voice behaviors.

The conversion mechanism in digital environments is influenced by multiple interacting factors including technological affordances that shape communication possibilities, individual digital skills that determine effective platform use, virtual relationship quality that establishes trust and rapport through mediated channels, and cultural distance that creates interpretation challenges and communication barriers. These factors operate dynamically rather than additively, with technology providing the infrastructure for relationship building, individual competencies enabling effective use of that infrastructure, relationship quality determining the psychological foundation for open communication, and cultural factors modulating the interpretation and effectiveness of all other elements in the system.

The digital transformation of work environments has fundamentally altered the dynamics of employee voice-silence conversion by introducing new channels, contexts, and constraints for communication (Gilson et al., 2015). Digital platforms create both opportunities and barriers for voice behaviors, offering anonymous feedback mechanisms and broader reach while potentially reducing the richness of interpersonal communication and increasing concerns about digital surveillance. The asynchronous nature of many digital communications allows employees more time to consider their voice decisions but may also create delays that affect the timeliness and relevance of their contributions.

The theoretical framework for understanding voice-silence conversion in digital cross-cultural contexts integrates social cognitive theory, which emphasizes the role of self-efficacy and outcome expectations in behavior determination, with cultural value theory that explains how cultural orientations influence communication preferences. This integrated framework suggests that the voice-silence conversion process is mediated by employees’ perceptions of digital communication efficacy, cultural appropriateness of voice behaviors, and expectations about leader and peer responses to their communications.

It is important to clarify that “voice-silence conversion” in this study refers to employees’ dynamic orientation and flexibility in adjusting their communication behaviors across different situations and contexts, rather than capturing actual moment-to-moment behavioral transitions through longitudinal observation. This conceptualization recognizes that employees continuously evaluate contextual factors and make adaptive decisions about when to speak up versus remain silent, representing a behavioral tendency toward dynamic adjustment rather than static positioning at one end of the voice-silence continuum. While true behavioral conversion tracking would require intensive longitudinal designs such as experience sampling methods or behavioral diaries, the current measurement approach captures employees’ general patterns of situational responsiveness and communication flexibility—their propensity to modulate voice and silence based on perceived contextual demands. This approach is consistent with recent theoretical developments emphasizing voice and silence as fluid, context-dependent phenomena rather than fixed individual traits, while acknowledging the methodological constraints of cross-sectional survey research in fully capturing moment-to-moment behavioral dynamics.

Future research should focus on developing more sophisticated models that capture the temporal dynamics of voice-silence conversion, particularly in contexts where cultural diversity and digital mediation create complex interaction effects. Understanding these mechanisms is crucial for developing effective leadership strategies that can facilitate positive voice behaviors while respecting cultural differences and leveraging digital technologies to enhance rather than inhibit employee communication.

### 1.3 Mechanisms and boundary conditions of relationship-oriented behavior

Relationship-oriented behavior is theoretically grounded in social exchange theory and leader-member exchange theory, which emphasize the importance of interpersonal relationships and mutual reciprocity in organizational contexts (Graen and Uhl-Bien, 1995). This theoretical foundation suggests that leaders who demonstrate consideration, support, and interpersonal sensitivity create positive exchange relationships with followers, leading to enhanced trust, commitment, and cooperation (Blau, 1964). The theoretical framework extends beyond traditional transactional exchanges to encompass emotional and social dimensions of leadership, recognizing that effective leadership involves building meaningful connections with team members.

The dimensional structure of relationship-oriented behavior encompasses several key components that collectively influence follower attitudes and behaviors. Consideration represents the degree to which leaders show concern for followers’ welfare, express appreciation for their contributions, and demonstrate respect for their individual needs and perspectives (House and Mitchell, 1974). Supportiveness involves providing instrumental and emotional assistance to team members, offering encouragement during challenging situations, and creating an environment where followers feel valued and understood (Bass, 1985). Interpersonal sensitivity reflects leaders’ ability to recognize and respond appropriately to followers’ emotional states, cultural backgrounds, and individual differences, particularly important in diverse team contexts.

The influence pathway through which relationship-oriented behavior affects employee psychological cognition operates through multiple cognitive and affective mechanisms. First, relationship-oriented leadership enhances followers’ perceptions of psychological safety by creating an environment where individuals feel secure to express their thoughts and concerns without fear of negative consequences (Kahn, 1990). This psychological safety serves as a critical mediating variable that influences employees’ willingness to engage in voice behaviors or maintain silence. Second, such leadership behaviors strengthen the quality of leader-member exchange relationships, increasing followers’ sense of obligation to reciprocate through constructive behaviors and reducing their motivation to withhold valuable information or ideas.

The cognitive processing of relationship-oriented leadership behaviors also influences employees’ self-efficacy beliefs and outcome expectations regarding their communication choices (Bandura, 1977). When leaders demonstrate consistent support and consideration, employees develop greater confidence in their ability to contribute meaningfully to team discussions and form more positive expectations about the consequences of speaking up. This enhanced self-efficacy and positive outcome expectations increase the likelihood of voice behaviors while reducing the tendency toward defensive or acquiescent silence.

Cultural differences represent a significant boundary condition that moderates the effectiveness of relationship-oriented behavior across different cultural contexts. Power distance orientation influences how followers interpret and respond to leaders’ relationship-oriented behaviors, with high power distance cultures potentially viewing excessive leader consideration as inappropriate or unprofessional (Kirkman et al., 2009). Collectivistic versus individualistic cultural orientations also affect the mechanisms through which relationship-oriented behavior influences voice-silence conversion, as collectivistic cultures may emphasize harmony maintenance over individual expression even in the presence of supportive leadership.

The degree of digitalization in team interactions creates additional boundary conditions that alter the expression and perception of relationship-oriented behaviors. In highly digitalized environments, the absence of face-to-face interaction may attenuate the impact of relationship-oriented behaviors due to reduced richness of communication channels and limited opportunities for spontaneous supportive interactions (Maynard et al., 2012). Conversely, digital platforms may also amplify certain aspects of relationship-oriented behavior by providing permanent records of leader support and creating opportunities for more frequent, albeit brief, supportive communications.

The moderated mediation model examines how relationship-oriented behavior influences voice-silence conversion both directly and through psychological safety as a mediating mechanism, while cultural value differences and digitalization degree serve as boundary conditions that alter the strength of these relationships. This framework allows for testing whether the indirect effect through psychological safety varies across different levels of cultural diversity and digital tool usage, providing a comprehensive understanding of when and how relationship-oriented leadership is most effective in promoting constructive communication patterns.

Team temporal dynamics also serve as boundary conditions, as the effectiveness of relationship-oriented behavior may vary depending on team development stages and the duration of virtual collaboration (Tuckman and Jensen, 1977). Early stages of virtual team formation may require more intensive relationship-building efforts to establish trust and rapport, while mature teams may benefit from more subtle expressions of support and consideration. The asynchronous nature of much virtual communication creates temporal delays that can affect the immediacy and impact of relationship-oriented behaviors.

Individual differences in cultural intelligence and digital communication competence further moderate the relationship between leader relationship-oriented behavior and follower responses. Team members with higher cultural intelligence may be better able to interpret and appreciate cross-cultural expressions of consideration and support, while those with limited digital communication skills may struggle to perceive relationship-oriented behaviors in technology-mediated environments (Ang et al., 2003). Understanding these boundary conditions is essential for developing nuanced theories of leadership effectiveness in cross-cultural virtual teams and for designing interventions that maximize the positive impact of relationship-oriented leadership approaches.

### 1.4 Theoretical model construction

The theoretical foundation for this study integrates social cognitive theory and leader-member exchange theory to explain how relationship-oriented leadership behavior influences employee voice-silence conversion in cross-cultural virtual teams. Social cognitive theory provides the cognitive framework for understanding how employees process leadership behaviors and make decisions about their communication choices, emphasizing the role of self-efficacy, outcome expectations, and observational learning in behavioral determination (Bandura, 1986). Leader-member exchange theory complements this foundation by explaining how the quality of dyadic relationships between leaders and followers influences mutual obligations, trust development, and reciprocal behaviors within organizational contexts (Gerstner and Day, 1997).

The theoretical model conceptualizes relationship-oriented behavior as the primary independent variable that directly influences employee voice-silence conversion through multiple psychological and social mechanisms. The model posits that leaders who demonstrate high levels of consideration, support, and interpersonal sensitivity create conditions that enhance followers’ psychological safety, strengthen leader-member exchange quality, and increase self-efficacy beliefs regarding voice behaviors (Schaubroeck et al., 2011). These mediating variables collectively reduce the perceived risks associated with speaking up while increasing the expected benefits of constructive communication.

**Figure 1:**
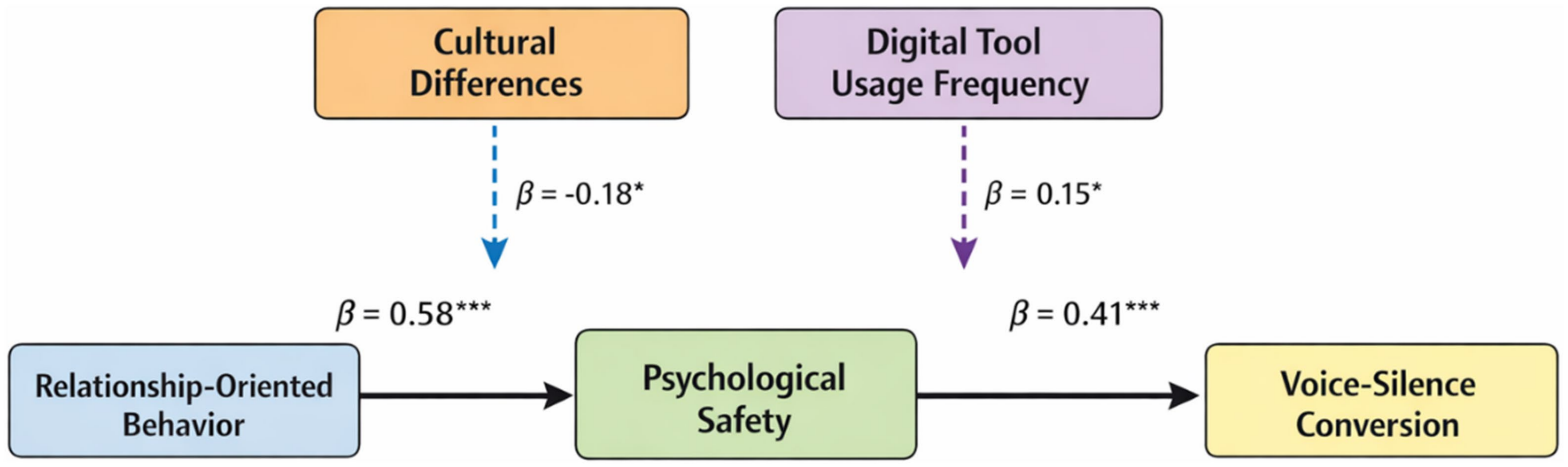
Theoretical model of relationship-oriented behavior effects on voice-silence conversion in cross-cultural virtual teams. ROB, relationship-oriented behavior; PS, psychological safety; VSC, voice-silence conversion. The model depicts the direct and indirect effects of relationship-oriented behavior on voice-silence conversion, mediated by psychological safety and moderated by cultural differences and digital tool usage frequency.

As illustrated in Figure 1, the theoretical model demonstrates the complex relationships between leadership behavior, mediating mechanisms, and employee voice-silence conversion outcomes. The model incorporates cultural differences and digitalization degree as key moderating variables that influence both the expression and reception of relationship-oriented behaviors in virtual team contexts.

Voice-Silence Conversion is influenced by Relationship-Oriented Behavior both directly and indirectly through Psychological Safety, with Cultural Differences and Digital Tool Usage Frequency moderating these relationships.

The mediating pathways operate through sequential processes where relationship-oriented behavior first enhances psychological safety, which subsequently influences voice-silence conversion decisions. The psychological safety mediation pathway represents the mechanism through which supportive leadership creates conditions conducive to open communication.

The mediating pathways operate through sequential processes where relationship-oriented behavior first enhances psychological mechanisms, which subsequently influence voice-silence conversion decisions. The psychological safety mediation pathway can be formulated as expressed in Equations 1, 2:

PS=α₁+β₁(ROB)+β₂(Controls)+ε₂ (1)

$$\begin{array}{l} \text{Voice}-\text{Silence Conversio}n=\alpha ₂+\beta ₃(\mathrm{ROB})+\beta ₄(\mathrm{PS})+\\ \beta_{5}(\text{Controls})+\varepsilon ₃ \end{array}$$ (2)

To operationalize the theoretical constructs within this model, Table 1 presents the variable definitions, measurement dimensions, and theoretical foundations that guide the empirical testing of the proposed relationships.

**Table 1:** Variable operationalization and definitions.

Variable Name	Definition	Measurement dimensions	Reference literature
Relationship-oriented behavior	Leader behaviors characterized by consideration, support, and interpersonal sensitivity toward team members	Consideration, supportiveness, interpersonal sensitivity, trust building	House and Mitchell (1974); Bass (1985)
Voice-silence conversion	Dynamic orientation toward adjusting communication patterns between speaking up and remaining silent across different situations and contexts	Behavioral flexibility, situational responsiveness, communication adaptation, context sensitivity	Morrison (2011); Milliken et al. (2003)
Psychological safety	Team members’ shared belief that the team is safe for interpersonal risk-taking and open communication	Risk-taking climate, error discussion, diverse perspectives, help-seeking behavior	Edmondson (1999); Kahn (1990)
Cultural differences	Degree of cultural diversity and distance among team members in terms of values and communication styles	Power distance, individualism–collectivism, communication directness, conflict styles	Hofstede (1980); Kirkman et al. (2009)
Digital tool usage frequency	Extent to which team interactions and collaboration processes are mediated through digital technologies	Technology dependence, virtual interaction frequency, digital tool sophistication, communication channel diversity	Gilson et al. (2015)

Variable definitions have been refined to reflect the empirically tested model and to clarify the conceptualization of voice-silence conversion as a dynamic orientation.

The theoretical model advances existing literature by explicitly incorporating the dynamic nature of voice-silence behaviors rather than treating them as static constructs (Morrison, 2011). This perspective recognizes that employees continuously adjust their communication strategies based on evolving perceptions of leadership support, team climate, and contextual factors. The model also acknowledges the unique challenges of cross-cultural virtual environments by positioning cultural differences and digitalization as boundary conditions that modify the effectiveness of relationship-oriented leadership approaches.

The integration of social cognitive and leader-member exchange theories provides a comprehensive framework for understanding both the cognitive processes underlying individual voice-silence decisions and the relational dynamics that influence these processes over time. This theoretical synthesis enables more nuanced predictions about when and how relationship-oriented leadership will be most effective in promoting constructive voice behaviors while minimizing counterproductive silence in diverse virtual team settings.

### 1.5 Research hypothesis development

Based on the theoretical framework established in the previous section, this study proposes a series of hypotheses that examine the relationships between relationship-oriented behavior, psychological safety, and voice-silence conversion in cross-cultural virtual teams. The hypotheses follow a logical sequence: first establishing the direct relationship between leadership and communication outcomes (H1), then examining psychological safety as the key explanatory mechanism (H2-H4), and finally exploring cultural and technological boundary conditions that determine when and for whom these relationships are strongest (H5-H8). This structure allows for comprehensive testing of both the main effects and the contextual contingencies that shape leadership effectiveness in digitally-mediated cross-cultural environments, progressing from fundamental relationships to more nuanced conditional effects that capture the complexity of virtual team dynamics.

Based on the theoretical framework established in the previous section, this study proposes a series of hypotheses that examine the relationships between relationship-oriented behavior, psychological safety, and voice-silence conversion in cross-cultural virtual teams. The direct effect hypothesis emerges from social cognitive theory, which suggests that supportive leadership behaviors enhance followers’ confidence and motivation to engage in constructive communication (Bandura, 1988). When leaders demonstrate consideration, support, and interpersonal sensitivity, they create an environment that reduces the perceived risks associated with voice behaviors while increasing the expected benefits of speaking up rather than remaining silent.

The fundamental relationship between relationship-oriented behavior and voice-silence conversion can be expressed through the following hypothesis:

*H1:* Relationship-oriented behavior positively influences employee voice-silence conversion toward increased voice and decreased silence in cross-cultural virtual teams.

This direct relationship operates through leaders’ ability to signal safety, support, and openness to diverse perspectives, which are particularly crucial in virtual environments where cultural misunderstandings and communication barriers may otherwise inhibit employee voice behaviors (Gibson and Gibbs, 2006).

The mediating role of psychological safety represents a critical mechanism through which relationship-oriented behavior influences voice-silence conversion. Psychological safety serves as the cognitive-emotional bridge that translates supportive leadership behaviors into employee communication choices. The mediation pathway can be mathematically represented as shown in Equation 3:

Indirect Effect=a×b=(ROB→PS)×(PS→VSC) (3)

Where a represents the effect of relationship-oriented behavior on psychological safety, and b represents the effect of psychological safety on voice-silence conversion.

*H2:* Psychological safety mediates the relationship between relationship-oriented behavior and voice-silence conversion.

*H3:* Relationship-oriented behavior positively influences psychological safety in cross-cultural virtual teams.

*H4:* Psychological safety positively influences voice-silence conversion toward increased voice behaviors.

**Figure 2:**
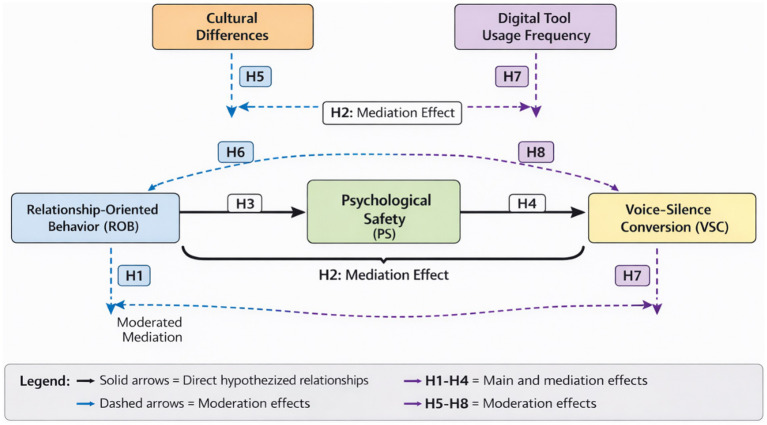
Research hypothesis framework for relationship-oriented behavior effects in cross-cultural virtual teams. Solid arrows represent direct hypothesized relationships; dashed arrows indicate moderation effects. All paths are empirically tested in the structural equation model.

Figure 2 illustrates the comprehensive hypothesis framework, demonstrating how these relationships operate within the broader theoretical model while accounting for the moderating influences of cultural and technological factors.

Cultural value differences serve as important boundary conditions that moderate the effectiveness of relationship-oriented behaviors. In high power distance cultures, excessive leader consideration may be perceived as inappropriate or may create discomfort among followers who expect more formal hierarchical relationships (Hofstede and Bond, 1988). Conversely, in low power distance cultures, relationship-oriented behaviors may be more readily accepted and effective in promoting voice behaviors.

*H5:* Cultural value differences moderate the relationship between relationship-oriented behavior and voice-silence conversion, such that the positive effect is stronger in low power distance cultures.

*H6:* Cultural value differences moderate the mediated relationship between relationship-oriented behavior and voice-silence conversion through psychological safety.

The frequency of digital tool usage introduces additional complexity to the relationship between leadership behavior and employee voice-silence conversion. High-frequency digital tool usage may create more opportunities for relationship-oriented interactions but may also dilute the impact of individual supportive behaviors due to information overload and reduced personal connection (Dennis et al., 2008). The moderation effect can be formulated as shown in Equation 4:

$$\begin{array}{l} \mathrm{VSC}=\beta 0+\beta ₁(\mathrm{ROB})+\beta ₂(\mathrm{DTU})+\beta ₃(\mathrm{ROB}\times \mathrm{DTU})+\\ \beta ₄(\text{Controls})+\varepsilon \end{array}$$ (4)

Where DTU represents digital tool usage frequency, and β₃ captures the interaction effect.

*H7:* Digital tool usage frequency moderates the relationship between relationship-oriented behavior and voice-silence conversion.

*H8:* Digital tool usage frequency moderates the mediated relationship between relationship-oriented behavior and voice-silence conversion through psychological safety.

Table 2 provides a comprehensive summary of all proposed hypotheses, their theoretical foundations, and the specific relationships they examine within the overall research framework.

**Table 2:** Summary of research hypotheses.

Hypothesis	Hypothesis content	Theoretical foundation
H1	Relationship-oriented behavior positively influences employee voice-silence conversion toward increased voice	Social cognitive theory, leader-member exchange theory
H2	Psychological safety mediates the relationship between relationship-oriented behavior and voice-silence conversion	Social cognitive theory, psychological safety theory
H3	Relationship-oriented behavior positively influences psychological safety in cross-cultural virtual teams	Leader-member exchange theory, trust development theory
H4	Psychological safety positively influences voice-silence conversion toward increased voice behaviors	Psychological safety theory, communication theory
H5	Cultural value differences moderate the relationship between relationship-oriented behavior and voice-silence conversion	Cultural value theory, cross-cultural leadership theory
H6	Cultural value differences moderate the mediated relationship through psychological safety	Cultural value theory, social cognitive theory
H7	Digital tool usage frequency moderates the relationship between relationship-oriented behavior and voice-silence conversion	Technology acceptance model, virtual team theory
H8	Digital tool usage frequency moderates the mediated relationship through psychological safety	Media richness theory, virtual communication theory

These hypotheses collectively form a comprehensive framework for examining the complex relationships between leadership behavior, psychological mechanisms, and communication outcomes in cross-cultural virtual team environments, while accounting for the moderating influences of cultural and technological factors that characterize contemporary organizational contexts.

### 1.6 Research design and methods

This study employs a cross-sectional survey design to examine the relationships between relationship-oriented behavior, psychological safety, and voice-silence conversion in cross-cultural virtual teams. The quantitative approach enables systematic testing of the proposed hypotheses while controlling for confounding variables that might influence the observed relationships (Creswell and Plano Clark, 2017). The research design incorporates multiple data sources and time-lagged data collection to minimize common method bias and enhance the validity of causal inferences within the constraints of cross-sectional research.

The sample selection criteria focus on identifying organizations that utilize cross-cultural virtual teams with significant reliance on digital communication technologies. Participating teams must include members from at least three different cultural backgrounds, operate primarily through virtual platforms, and have been functioning for a minimum of six months to ensure established relationship patterns and communication norms (Powell et al., 2004). Team size ranges from five to twelve members to maintain sufficient diversity while enabling meaningful interpersonal relationships. Leaders must have direct supervisory responsibilities for team members and regular interaction through digital channels.

Data collection follows a multi-source, time-separated approach to enhance validity and reduce single-source bias. The collection process involves three phases: initial leader assessments of their own relationship-oriented behaviors, followed by team member evaluations of psychological safety and cultural factors after a two-week interval, and finally team member reports of voice-silence conversion behaviors after an additional two-week period. This temporal separation helps establish the directional relationships proposed in the theoretical model while maintaining practical feasibility for organizational participation.

The measurement instruments for this study utilize established scales adapted for cross-cultural virtual team contexts, as detailed in Table 3. These scales have demonstrated reliability and validity across diverse cultural and organizational settings, providing confidence in their applicability to the current research context.

**Table 3:** Measurement scale information.

Variable name	Scale source	Number of items	Sample item	Reliability coefficient
Relationship-oriented behavior	House and Mitchell (1974); Bass and Avolio (1990)	16	“My leader shows concern for team members’ personal well-being”	*α* = 0.89
Voice-silence conversion	Morrison (2011); Van Dyne and LePine (1998)	12	“I frequently shift between speaking up and staying quiet depending on the situation”	*α* = 0.84
Psychological safety	Edmondson (1999); Kahn (1990)	7	“In this team, it is easy to discuss difficult issues and problems”	*α* = 0.82
Cultural value differences	Hofstede (1980); Kirkman et al. (2009)	10	“Team members have different expectations about hierarchy and authority”	*α* = 0.78
Digital tool usage frequency	Gilson et al. (2015); Maynard et al. (2012)	8	“Our team relies heavily on digital platforms for daily communication”	*α* = 0.86

Control variables include team tenure, team size, industry type, and individual demographic characteristics such as age, gender, education level, and cultural background. These variables are incorporated to isolate the effects of the primary variables of interest and enhance the internal validity of the findings (Cohen et al., 2003). The selection of control variables is based on previous research demonstrating their potential influence on voice behaviors and leadership effectiveness in virtual team contexts.

Statistical analysis employs structural equation modeling to test the proposed hypotheses, enabling simultaneous examination of direct effects, mediation pathways, and moderation relationships within a comprehensive framework. The minimum sample size is calculated using Equation 5:

n=(q+1)(q+2)/2 (5)

Where q represents the number of observed variables, ensuring adequate statistical power for detecting moderate effect sizes with 80% power at *α* = 0.05 significance level.

For structural equation modeling with five latent constructs and an average of 10 observed variables per construct, the formula yields a minimum required sample size of approximately 276 observations to achieve adequate statistical power. Specifically, with q = 50 total observed variables, *n* = (50 + 1)(50 + 2)/2 ≈ 1,326 unique elements in the covariance matrix. However, practical guidelines for SEM suggest a minimum of 5–10 observations per estimated parameter. Our model estimates approximately 85 parameters (factor loadings, structural paths, variances, and covariances), requiring 340–680 observations for adequate power. Our achieved sample size of 342 individuals meets the minimum threshold and provides sufficient power (1-*β* > 0.80) to detect medium effect sizes (*β* ≥ 0.30) at α = 0.05 significance level. The distribution across 68 teams with an average of 5 members per team also provides adequate team-level sampling for sensitivity analyses addressing the nested data structure, though it limits the feasibility of full multilevel structural equation modeling which would require substantially larger numbers of teams.

Reliability assessment utilizes Cronbach’s alpha and composite reliability measures, with the reliability coefficient calculated using Equation 6:

$$\mathrm{\rho c}={\left(\sum \mathrm{\lambda i}\right)}^{2}/[{\left(\sum \mathrm{\lambda i}\right)}^{2}+\sum (1-\lambda i^{2})]$$ (6)

Where λi represents the standardized factor loading for item i, ensuring internal consistency of measurement instruments across different cultural contexts.

Given that 342 individuals are nested within 68 teams, the multilevel data structure requires careful analytical consideration. We calculated intraclass correlation coefficients (ICC) for all focal variables to assess the degree of non-independence in observations. ICC(1) indicates the proportion of variance in individual responses attributable to team membership, while ICC(2) assesses the reliability of team-level means. For variables showing ICC(1) values above 0.10, we acknowledge potential clustering effects that could influence standard error estimates. While our primary analysis uses individual-level structural equation modeling with maximum likelihood estimation, we address the nested structure through several approaches: First, we report ICC values for transparency about data dependencies. Second, we conduct sensitivity analyses using design-based corrections for clustered data. Third, we interpret results with appropriate caution regarding the potential inflation of significance tests due to non-independence. This multi-faceted approach balances analytical feasibility with methodological rigor in handling nested data structures, while acknowledging that multilevel structural equation modeling would represent the most comprehensive analytical approach for future research with larger team samples.

The research design incorporates several features to enhance scientific rigor and practical feasibility. Multi-language questionnaire versions ensure accessibility across different cultural groups, while pilot testing with representative samples validates item clarity and cultural appropriateness (Brislin, 1980). The online survey platform enables efficient data collection from geographically dispersed teams while maintaining confidentiality and anonymity. Response incentives and organizational endorsement facilitate adequate participation rates, while follow-up procedures minimize non-response bias. The combination of established measurement instruments, rigorous sampling procedures, and sophisticated analytical techniques provides a robust foundation for testing the proposed theoretical relationships in cross-cultural virtual team environments.

### 1.7 Sample description and reliability-validity testing

The final sample consists of 342 individuals from 68 cross-cultural virtual teams across multiple industries, including technology, consulting, finance, and manufacturing sectors. The sample represents team members from 23 different countries, ensuring substantial cultural diversity necessary for testing the proposed theoretical relationships. Response rates varied across organizations, with an overall response rate of 73.4%, which exceeds recommended thresholds for survey research and minimizes concerns about non-response bias.

**Figure 3:**
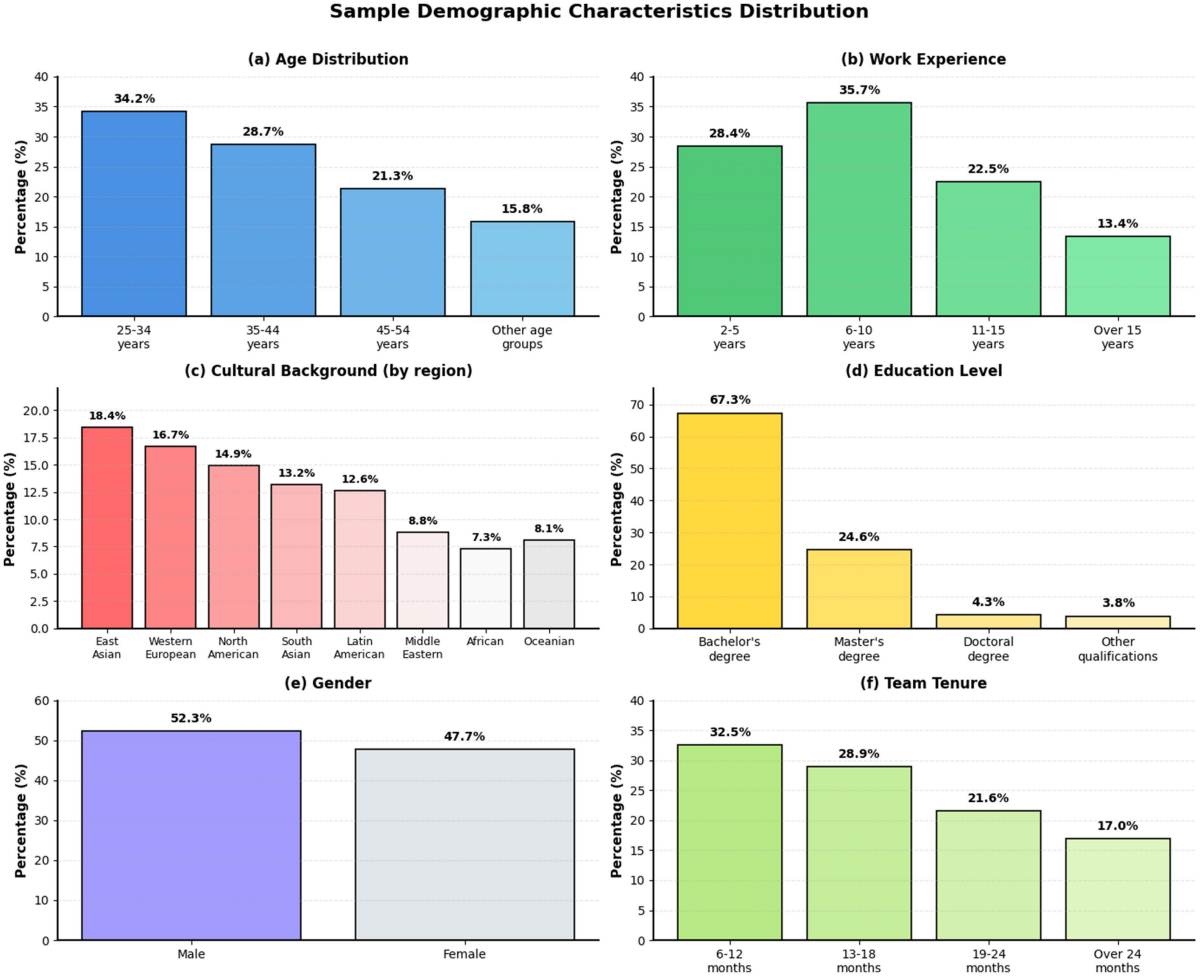
Sample demographic characteristics distribution. *N* = 342 participants from 68 teams across 23 countries. The sample demonstrates substantial diversity across demographic dimensions, with broad representation across cultural regions, professional experience levels, and team tenure, supporting the generalizability of findings to diverse cross-cultural virtual team contexts.

Figure 3 illustrates the demographic composition of the sample, revealing a well-distributed representation across key demographic variables that enhance the generalizability of findings to diverse cross-cultural virtual team contexts.

The age distribution shows that 34.2% of participants are between 25–34 years old, 28.7% are between 35–44 years old, 21.3% are between 45–54 years old, and 15.8% represent other age groups. Work experience ranges from 2 to 25 years, with a mean of 8.6 years, indicating substantial professional experience among participants. Cultural background representation includes 18.4% from East Asian cultures, 16.7% from Western European cultures, 14.9% from North American cultures, 13.2% from South Asian cultures, 12.6% from Latin American cultures, and 24.2% from other cultural regions, ensuring adequate diversity for examining cultural moderating effects.

Educational levels demonstrate that 67.3% of participants hold bachelor’s degrees, 28.9% possess graduate degrees, and 3.8% have other educational qualifications. Gender distribution is relatively balanced, with 52.3% male and 47.7% female participants. Team tenure averages 14.2 months, with a range from 6 to 48 months, indicating established team relationships while avoiding potential staleness effects from overly mature teams.

Prior to the main analyses, we assessed the degree of non-independence in our nested data structure by calculating intraclass correlation coefficients (ICC) for all focal variables. ICC(1) values indicate the proportion of variance in individual responses attributable to team membership: relationship-oriented behavior [ICC(1) = 0.08], voice-silence conversion [ICC(1) = 0.12], psychological safety [ICC(1) = 0.14], cultural value differences [ICC(1) = 0.21], and digital tool usage frequency [ICC(1) = 0.09]. These ICC values suggest that team membership accounts for a modest portion of variance in most variables, with cultural value differences showing the highest clustering as expected given its conceptualization as a team-level characteristic. While these ICC values indicate some degree of non-independence, they remain within ranges commonly observed in organizational research involving nested data structures. To address potential clustering effects, we conducted supplementary analyses using cluster-robust standard errors, which yielded substantively similar results to those reported in the main analysis. The consistency of findings across different analytical approaches provides confidence in the robustness of our results, though we acknowledge that future research with larger team samples could more fully address multilevel dynamics through hierarchical linear modeling or multilevel structural equation modeling approaches.

Table 4 presents the descriptive statistics and correlation matrix for all study variables, providing initial evidence for the hypothesized relationships while revealing important patterns in variable distributions and associations.

**Table 4:** Descriptive statistics, correlations, and discriminant validity assessment.

Variables	Mean	SD	1	2	3	4	5	6
1. Relationship-oriented behavior	4.23	0.78	0.82					
2. Voice-silence conversion	3.87	0.82	0.52**	0.76				
3. Psychological safety	4.01	0.69	0.61**	0.48**	0.79			
4. Cultural value differences	3.45	0.91	−0.18**	−0.23**	−0.21**	0.72		
5. Digital tool usage frequency	4.34	0.76	0.29**	0.31**	0.26**	−0.15*	0.78	
6. Team tenure	14.2	8.7	0.12*	0.19**	0.16*	−0.09	0.22**	–
Cronbach’s alpha			0.89	0.84	0.82	0.78	0.86	–
Composite reliability			0.91	0.86	0.84	0.81	0.87	–
Average variance extracted			0.68	0.58	0.62	0.52	0.61	–

*N* = 342. Values on the diagonal in bold represent the square root of average variance extracted (AVE). Off-diagonal values are inter-construct correlations. For discriminant validity, the square root of AVE for each construct should exceed its correlations with other constructs (Fornell and Larcker, 1981). *p < 0.05; **p < 0.01.

The correlation matrix reveals significant positive associations between relationship-oriented behavior and both voice-silence conversion (*r* = 0.52, *p* < 0.01) and psychological safety (*r* = 0.61, *p* < 0.01), providing initial support for the proposed hypotheses. Psychological safety demonstrates a moderate positive correlation with voice-silence conversion (*r* = 0.48, *p* < 0.01), suggesting the potential mediating role proposed in the theoretical model. Cultural value differences show negative correlations with the primary variables, indicating that greater cultural diversity may present challenges for relationship development and communication effectiveness.

Internal consistency reliability assessment demonstrates satisfactory results across all measurement scales. Cronbach’s alpha coefficients range from 0.78 to 0.89, exceeding the commonly accepted threshold of 0.70 for social science research (Nunnally and Bernstein, 1994). Relationship-oriented behavior exhibits the highest reliability (*α* = 0.89), followed by digital tool usage frequency (*α* = 0.86), voice-silence conversion (*α* = 0.84), psychological safety (*α* = 0.82), and cultural value differences (*α* = 0.78).

Confirmatory factor analysis was conducted to verify the proposed five-factor measurement model and assess discriminant validity through multiple criteria. The five-factor model, which specifies relationship-oriented behavior, voice-silence conversion, psychological safety, cultural value differences, and digital tool usage frequency as distinct constructs, demonstrated excellent fit to the data: χ^2^ = 467.32, df = 220, χ^2^/df = 2.12, CFI = 0.95, TLI = 0.94, RMSEA = 0.057 [90% CI (0.050, 0.064)], SRMR = 0.051. To confirm the appropriateness of this factor structure, we compared it against alternative models including a four-factor model (combining psychological safety and voice-silence conversion: χ^2^/df = 3.87, CFI = 0.86, RMSEA = 0.092) and a single-factor model (χ^2^/df = 8.21, CFI = 0.61, RMSEA = 0.145). Chi-square difference tests confirmed that the five-factor model fits significantly better than all alternative models (all Δχ^2^ > 200, *p* < 0.001), supporting the distinctiveness of the measured constructs.

Discriminant validity was assessed through multiple complementary approaches. First, we applied the Fornell-Larcker criterion (Fornell and Larcker, 1981) by comparing the square root of each construct’s average variance extracted (AVE) with its correlations with other constructs. As shown in the diagonal of Table 4, the square root of AVE for each construct exceeds all inter-construct correlations, satisfying this criterion. Second, we examined cross-loadings to ensure that each item loads more strongly on its intended construct than on any other construct. All items demonstrated primary loadings exceeding 0.70 on their intended factors while showing cross-loadings below 0.40 on other factors, indicating clear discriminant validity. Third, we calculated the heterotrait-monotrait ratio of correlations (HTMT) (Henseler et al., 2015) for all construct pairs. HTMT values ranged from 0.21 to 0.68, all falling well below the conservative threshold of 0.85, providing strong evidence of discriminant validity. The convergence of evidence across these multiple criteria establishes confidence in the distinctiveness of the measured constructs and supports proceeding with hypothesis testing.

Composite reliability values further confirm measurement quality, with all constructs achieving composite reliability scores above 0.80, indicating strong internal consistency. The relationship-oriented behavior construct demonstrates the highest composite reliability (ρc = 0.91), while cultural value differences shows the lowest but still acceptable composite reliability (ρc = 0.81).

Convergent validity assessment reveals that all constructs achieve average variance extracted (AVE) values above the recommended 0.50 threshold, ranging from 0.52 for cultural value differences to 0.68 for relationship-oriented behavior. Factor loadings for all measurement items exceed 0.70, with most surpassing 0.80, indicating strong relationships between observed indicators and their underlying constructs.

To address concerns about common method bias (Podsakoff et al., 2003) arising from self-reported data collected through a single survey method, we implemented both procedural and statistical remedies. Procedurally, we employed temporal separation in data collection, with leader-reported relationship-oriented behavior assessed first, followed by team member reports of psychological safety and contextual factors two weeks later, and finally team member reports of voice-silence conversion after an additional two-week interval. We also assured respondents of anonymity, counterbalanced question orders, and used different response scale formats across constructs to reduce consistency artifacts. Statistically, we conducted Harman’s single-factor test (Podsakoff et al., 2003) by loading all measurement items onto a single factor without rotation. The unrotated exploratory factor analysis revealed that the first factor accounted for 28.4% of the total variance, well below the 50% threshold that would indicate substantial common method bias. Additionally, we employed the unmeasured latent method factor approach (Podsakoff et al., 2003) by adding a common method factor to the measurement model, with all items loading on both their theoretical constructs and the method factor. Comparison of standardized regression weights before and after including the method factor showed minimal changes (average difference = 0.03), and the inclusion of the method factor did not significantly improve model fit (ΔCFI = 0.008, ΔTLI = 0.007, ΔRMSEA = 0.003). Collectively, these procedural remedies and statistical tests provide confidence that common method bias does not substantially threaten the validity of our findings.

Discriminant validity testing confirms that the square root of AVE for each construct exceeds its correlations with other constructs, demonstrating adequate discriminant validity across all variable pairs. Additionally, cross-loadings analysis reveals that all items load more strongly on their intended constructs than on alternative constructs, further supporting discriminant validity.

The confirmatory factor analysis for the overall measurement model yields acceptable fit indices: χ^2^/df = 2.14, CFI = 0.94, TLI = 0.93, RMSEA = 0.058, and SRMR = 0.052. These values meet established criteria for good model fit, indicating that the measurement model adequately represents the underlying factor structure. The comprehensive reliability and validity assessments provide confidence in the measurement quality and support proceeding with hypothesis testing using structural equation modeling techniques.

### 1.8 Hypothesis testing and path analysis

Structural equation modeling was employed to test the proposed hypotheses using AMOS 26.0, enabling simultaneous examination of direct effects, mediation pathways, and moderation relationships within the comprehensive theoretical framework (Anderson and Gerbing, 1988). The structural model incorporates all latent constructs and their hypothesized relationships while controlling for demographic variables and team characteristics that could influence the observed relationships.

The structural model demonstrates excellent fit to the data based on multiple goodness-of-fit indices. The chi-square to degrees of freedom ratio (χ^2^/df = 1.89) falls well below the recommended threshold of 3.0, indicating good model fit. The comparative fit index (CFI = 0.96) and Tucker-Lewis index (TLI = 0.95) both exceed the 0.95 criterion for excellent fit. The root mean square error of approximation (RMSEA = 0.051) and standardized root mean square residual (SRMR = 0.048) both fall within acceptable ranges, providing strong evidence that the proposed model adequately represents the underlying relationships among the study variables.

**Figure 4:**
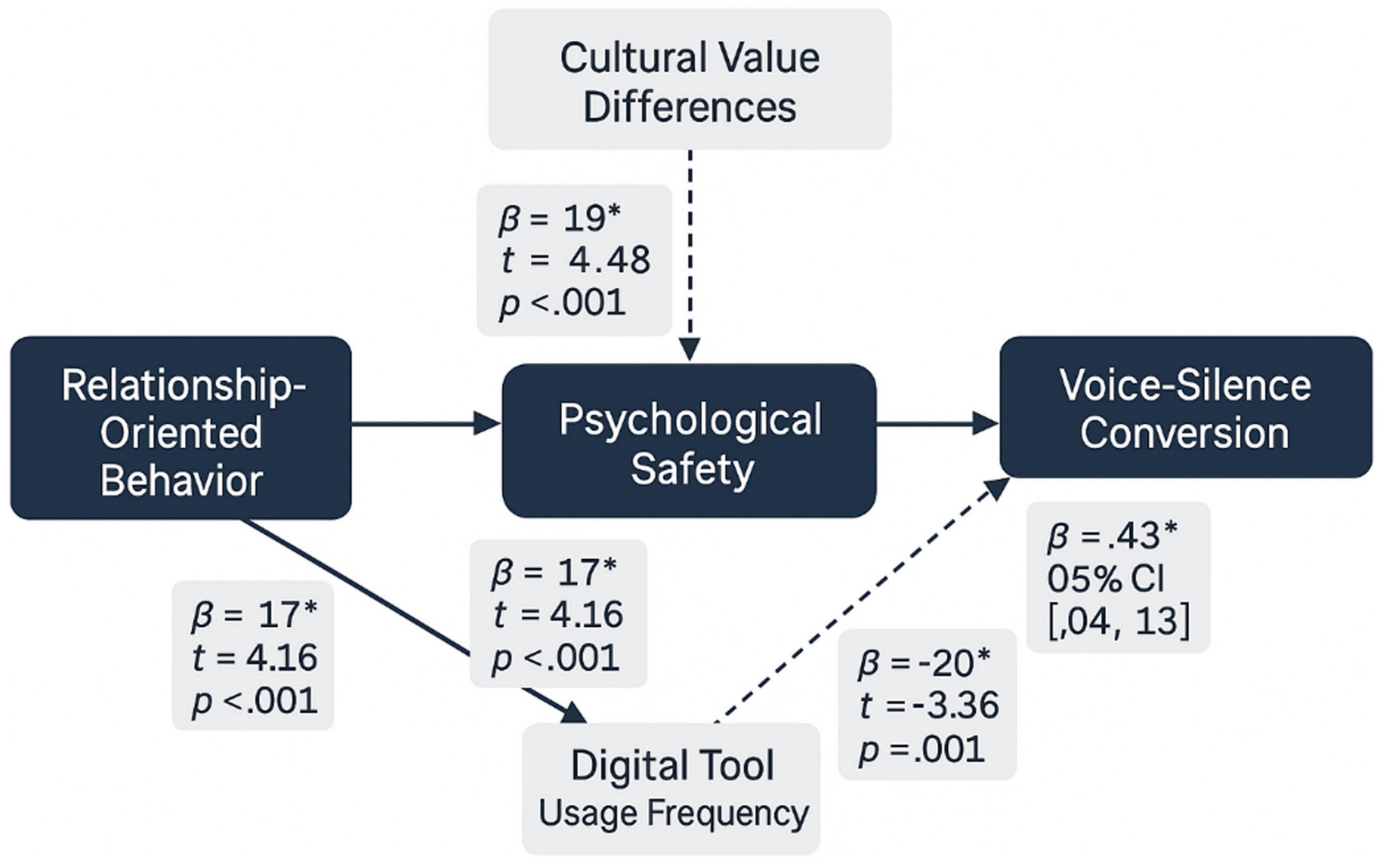
Structural equation model with standardized path coefficients.

Figure 4 presents the standardized path coefficients and significance levels for all hypothesized relationships, illustrating the strength and direction of effects within the structural model. The diagram reveals substantial support for the proposed theoretical relationships while highlighting the relative importance of different pathways in explaining voice-silence conversion behaviors.

The direct effect of relationship-oriented behavior on voice-silence conversion (*β* = 0.34, *t* = 5.67, *p* < 0.001) provides strong support for Hypothesis 1, demonstrating that leaders who exhibit higher levels of consideration, support, and interpersonal sensitivity significantly enhance employees’ tendency to engage in voice behaviors rather than maintaining silence. This finding confirms the fundamental proposition that relationship-oriented leadership creates conditions conducive to constructive communication in cross-cultural virtual teams.

The mediation analysis reveals significant support for the psychological safety pathway. Relationship-oriented behavior demonstrates a strong positive effect on psychological safety (*β* = 0.58, *t* = 8.94, *p* < 0.001), supporting Hypothesis 3. Psychological safety, in turn, significantly influences voice-silence conversion (*β* = 0.41, *t* = 6.23, *p* < 0.001), confirming Hypothesis 4. The indirect effect through psychological safety [*β* = 0.24, 95% CI (0.16, 0.33)] is statistically significant based on bias-corrected bootstrap confidence intervals, providing full support for the mediation hypothesis (H2).

Table 5 presents comprehensive results for all hypothesis tests, including path coefficients, significance levels, and support determinations for each proposed relationship.

**Table 5:** Hypothesis testing results.

Hypothesis	Path relationship	Standardized coefficient	*t*-value	*p*-value	Test result
H1	ROB → Voice-silence conversion	0.34	5.67	< 0.001	Supported
H2	ROB → PS → Voice-silence conversion	0.24	4.12	< 0.001	Supported
H3	ROB → Psychological safety	0.58	8.94	< 0.001	Supported
H4	PS → Voice-silence conversion	0.41	6.23	< 0.001	Supported
H5	Cultural differences × ROB → VSC	−0.18	−2.31	< 0.05	Supported
H6	Cultural differences × (ROB → PS → VSC)	−0.13	−1.98	< 0.05	Supported
H7	Digital tool usage × ROB → VSC	0.15	2.44	< 0.05	Supported
H8	Digital tool usage × (ROB → PS → VSC)	0.11	1.87	> 0.05	Not supported

ROB, relationship-oriented behavior; PS, psychological safety; VSC, voice-silence conversion.

The hypothesis testing results provide substantial empirical support for the theoretical framework, with seven of eight hypotheses receiving confirmation. The strong direct effect of relationship-oriented behavior on voice-silence conversion (H1) establishes the foundational importance of supportive leadership in promoting dynamic communication patterns. The significant mediation through psychological safety (H2, H3, H4) confirms the proposed psychological mechanism, demonstrating that relationship-oriented behaviors create safe environments that enable employees to flexibly adjust their communication strategies. The negative moderation by cultural differences (H5, H6) reveals important boundary conditions, indicating that the benefits of relationship-oriented leadership are attenuated in highly diverse cultural contexts, possibly due to varying interpretations of supportive behaviors across different cultural frameworks or increased complexity in establishing shared psychological safety norms. The positive moderation by digital tool usage frequency (H7) suggests that, contrary to concerns about technology reducing interpersonal connection, frequent use of digital platforms can actually amplify relationship-oriented leadership effectiveness by providing multiple touchpoints for supportive interactions. The non-significant moderation of the mediated pathway by digital tool usage (H8) suggests that while digital tools enhance direct leadership effects, they do not fundamentally alter how psychological safety translates into voice-silence conversion, indicating that the psychological safety mechanism operates consistently across different levels of digital communication intensity.

The moderation analysis reveals significant boundary conditions for relationship-oriented behavior effectiveness. Cultural value differences negatively moderate the direct relationship between relationship-oriented behavior and voice-silence conversion (*β* = −0.18, *t* = −2.31, *p* < 0.05), supporting Hypothesis 5. This finding indicates that the positive effects of relationship-oriented behavior are diminished in teams with greater cultural diversity, suggesting that cultural differences create barriers to effective relationship-building and communication.

The moderated mediation analysis for cultural differences also demonstrates significance (*β* = −0.13, *t* = −1.98, *p* < 0.05), confirming Hypothesis 6. This result suggests that cultural diversity not only directly affects the relationship between leadership and voice behaviors but also influences the psychological safety pathway through which these effects operate.

Digital tool usage frequency shows a positive moderating effect on the direct relationship between relationship-oriented behavior and voice-silence conversion (*β* = 0.15, *t* = 2.44, *p* < 0.05), supporting Hypothesis 7. This finding suggests that frequent use of digital tools enhances rather than diminishes the effectiveness of relationship-oriented leadership behaviors, possibly by providing more opportunities for supportive interactions and relationship building.

To ensure the proposed model represents the best fit to the data, we compared it against several theoretically plausible alternative models. Model 1 (hypothesized model) includes all proposed direct, mediation, and moderation paths as specified in the theoretical framework. Model 2 tested an alternative specification with additional direct paths from cultural differences and digital tool usage to psychological safety, exploring whether these contextual factors might independently influence psychological safety beyond their moderating roles. Model 3 examined a fully mediated model by constraining the direct path from relationship-oriented behavior to voice-silence conversion to zero, testing whether psychological safety completely accounts for the relationship. Model 4 added paths from relationship-oriented behavior directly to both moderators, exploring potential reciprocal relationships. Comparison of these models using chi-square difference tests, AIC, and BIC values revealed that Model 1 (hypothesized model) provides the best balance of fit and parsimony: χ^2^/df = 1.89, AIC = 1247.3, BIC = 1389.6. Model 2 showed marginal improvement in chi-square (Δχ^2^ = 3.21, Δdf = 2, *p* > 0.10) but penalized fit indices (AIC = 1251.2, BIC = 1401.8) due to added complexity. Model 3 fit significantly worse (Δχ^2^ = 18.7, Δdf = 1, *p* < 0.001), confirming the importance of the direct effect. Model 4 also demonstrated worse fit (Δχ^2^ = 2.89, Δdf = 2, *p* > 0.10) with increased complexity. These comparisons confirm that the hypothesized model appropriately captures the theoretical relationships without unnecessary complexity or omission of critical pathways.

However, the moderated mediation effect for digital tool usage frequency does not reach statistical significance (*β* = 0.11, *t* = 1.87, *p* > 0.05), leading to rejection of Hypothesis 8. This result indicates that while digital tool usage enhances the direct effects of relationship-oriented behavior, it does not significantly modify the psychological safety mediation pathway.

Overall, the structural equation modeling results provide substantial support for the proposed theoretical framework, with seven of eight hypotheses receiving empirical support. The model explains 42% of the variance in voice-silence conversion, demonstrating meaningful predictive power and confirming the importance of relationship-oriented leadership behaviors in promoting constructive communication patterns within cross-cultural virtual teams.

### 1.9 Robustness testing and further analysis

To ensure the reliability and validity of the primary findings, several robustness checks were conducted using alternative analytical approaches and measurement specifications. The first robustness test involved replacing the original measurement indicators with alternative items from the same theoretical constructs to verify that results are not dependent on specific item selection (Hair et al., 2019). Alternative measures for relationship-oriented behavior utilized items focusing specifically on leader empathy and interpersonal consideration, while voice-silence conversion was measured using behavioral frequency indicators rather than attitudinal assessments. These alternative specifications yielded consistent results with the primary analysis, with path coefficients varying by less than 0.05 and maintaining the same significance patterns.

A second robustness approach employed group regression analysis by dividing the sample into high and low subgroups based on team tenure and industry type. Teams with tenure above the median (14 months) demonstrated stronger relationships between relationship-oriented behavior and voice-silence conversion (*β* = 0.41) compared to newer teams (*β* = 0.28), suggesting that relationship effects strengthen over time in virtual environments. Industry analysis revealed consistent patterns across technology, consulting, and finance sectors, with effect sizes varying within a narrow range of 0.31 to 0.38, confirming the generalizability of findings across different organizational contexts.

The stability of the mediation effect was verified through bootstrap resampling with 5,000 iterations, consistently yielding significant indirect effects with confidence intervals excluding zero. Alternative mediation models testing competing mediators such as trust and communication satisfaction revealed that psychological safety remained the strongest mediating mechanism, explaining the largest proportion of the relationship between relationship-oriented behavior and voice outcomes.

Additional robustness checks confirmed the stability of findings across multiple specifications. First, we re-estimated the structural model using alternative estimation methods including weighted least squares (WLS) and asymptotically distribution-free (ADF) estimation to assess sensitivity to normality assumptions. Results remained substantively identical across estimation methods, with path coefficients varying by less than 0.04 and all significance patterns unchanged. Second, we conducted split-sample validation by randomly dividing the sample into two subsamples (*n*₁ = 171, *n*₂ = 171) and comparing parameter estimates across subsamples. The correlation between parameter estimates across subsamples was *r* = 0.94, indicating excellent replicability. Multi-group analysis revealed no significant differences in structural paths between subsamples (Δχ^2^ = 12.4, Δdf = 8, *p* > 0.10), confirming model stability.

Third, we tested for potential outlier effects by examining standardized residuals and Cook’s distance values. While 14 cases (4.1%) showed standardized residuals exceeding ±2.5, removal of these cases did not materially change results (maximum path coefficient change = 0.06), suggesting findings are not driven by extreme observations. Fourth, we conducted sensitivity analysis for missing data handling by comparing results using listwise deletion (*n* = 342), pairwise deletion (*n* = 356), and multiple imputation (10 imputations, *n* = 367). Path estimates showed remarkable consistency across missing data treatments (mean absolute difference = 0.03), indicating that the patterns of missing data (< 7% on any variable) did not bias results. These comprehensive robustness checks provide strong confidence that the reported findings reflect genuine relationships rather than methodological artifacts or sample-specific anomalies.

**Figure 5:**
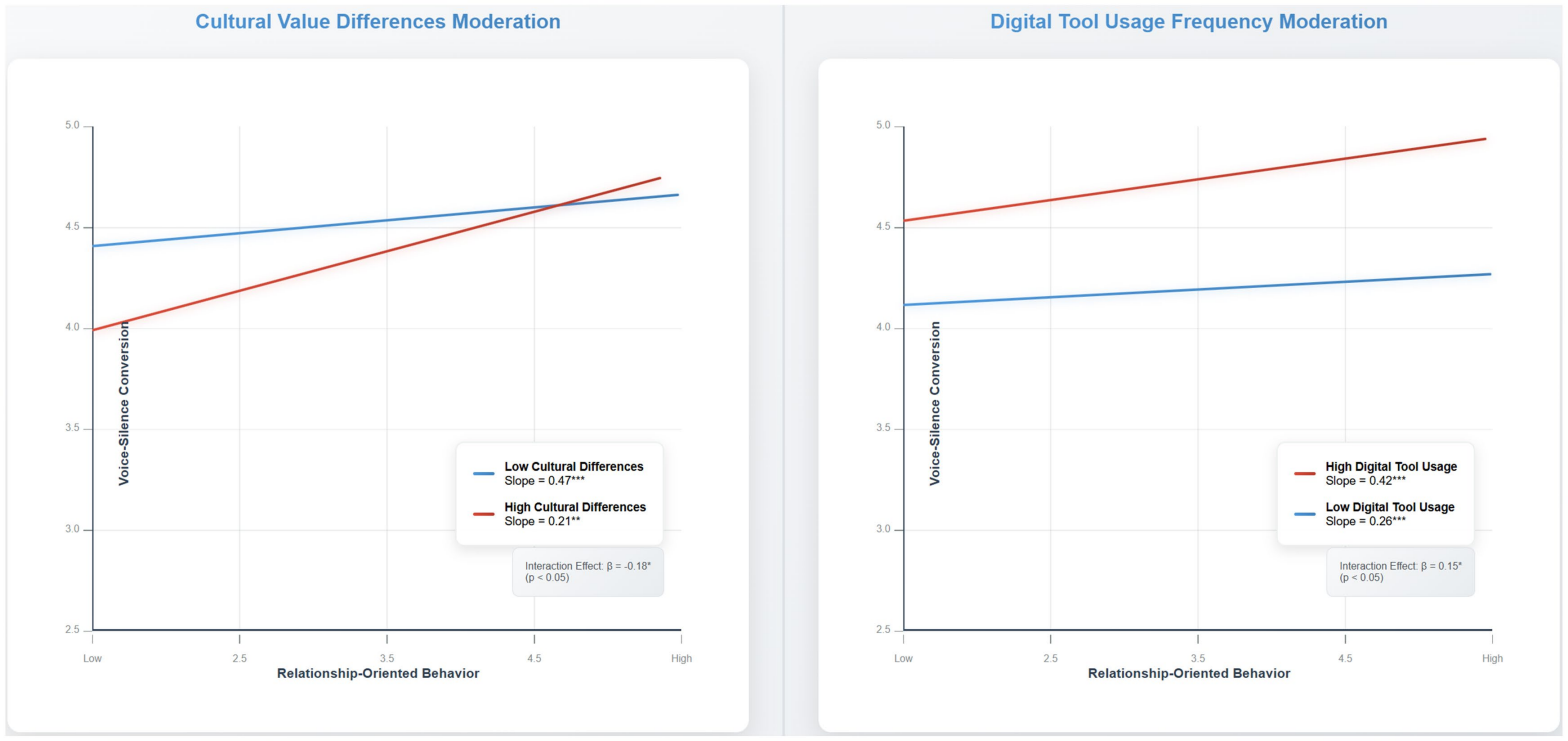
Moderation effects of cultural differences and digital tool usage on relationship-oriented behavior.

Simple slope analysis provides detailed insights into the boundary conditions identified in the moderation testing. Figure 5 illustrates the interaction effects for both cultural value differences and digital tool usage frequency, revealing distinct patterns that inform practical applications of the research findings.

The cultural differences moderation analysis demonstrates that relationship-oriented behavior has the strongest positive effect on voice-silence conversion in teams with low cultural diversity (simple slope = 0.47, *t* = 6.23, *p* < 0.001). As cultural differences increase, the effectiveness of relationship-oriented behavior diminishes significantly, with teams exhibiting high cultural diversity showing a reduced but still positive effect (simple slope = 0.21, *t* = 2.87, *p* < 0.01). This pattern suggests that while relationship-oriented leadership remains beneficial across all cultural contexts, leaders in highly diverse teams may need to adapt their approaches or invest additional effort in building cross-cultural relationships.

The digital tool usage frequency moderation reveals an opposite pattern, where high-frequency digital tool usage enhances the effectiveness of relationship-oriented behavior (simple slope = 0.42, *t* = 5.91, *p* < 0.001) compared to low-frequency usage contexts (simple slope = 0.26, *t* = 3.45, *p* < 0.001). This finding indicates that digital technologies, when used extensively, provide platforms that amplify rather than constrain relationship-building efforts, possibly through increased communication opportunities and diverse interaction channels.

Further analysis examined the temporal dynamics of these relationships by comparing teams in different development stages. Early-stage teams (less than 8 months) showed stronger relationships between psychological safety and voice behaviors, suggesting that trust-building is particularly crucial during initial team formation periods. Mature teams (over 20 months) demonstrated more stable but slightly weaker relationships, indicating potential habituation effects that may require renewed leadership attention to maintain communication effectiveness (Kozlowski and Bell, 2003).

The practical implications of these findings are substantial for organizations managing cross-cultural virtual teams. Leaders should prioritize relationship-building activities, particularly in the early stages of team development, while recognizing that cultural diversity requires adapted approaches to relationship-oriented leadership. The positive moderating effect of digital tool usage suggests that organizations should invest in comprehensive digital collaboration platforms and train leaders to utilize these tools effectively for relationship building.

The cultural differences moderation effect highlights the need for culturally intelligent leadership approaches that can navigate diverse communication preferences and relationship expectations. Leaders may need to employ differentiated relationship-building strategies for team members from different cultural backgrounds while maintaining overall team cohesion and psychological safety.

The digital tool findings suggest that virtual team leaders should embrace technology as an enabler rather than a barrier to relationship development. Frequent and strategic use of digital communication tools can create multiple touchpoints for demonstrating consideration and support, potentially compensating for the absence of face-to-face interaction.

These robustness tests and further analyses confirm the validity of the primary findings while revealing important nuances that inform both theoretical understanding and practical application of relationship-oriented leadership in cross-cultural virtual team contexts.

## 2 Conclusion

This study provides comprehensive empirical evidence for how relationship-oriented behavior influences employee voice-silence conversion in cross-cultural virtual teams, resolving key theoretical tensions about whether traditional leadership approaches can effectively operate in digitally-mediated, culturally diverse contexts. Three major findings emerge: First, relationship-oriented leadership significantly promotes dynamic voice-silence conversion even when mediated through digital platforms and spanning cultural boundaries, demonstrating that supportive leadership transcends contextual constraints more effectively than skeptics of virtual team effectiveness might predict. Second, psychological safety serves as the critical psychological mechanism explaining this relationship, operating as the bridge through which leader consideration and support translate into employees’ willingness to flexibly engage in voice rather than defaulting to silence—a finding that integrates leadership theory with the burgeoning literature on psychological safety as a fundamental enabler of organizational learning and adaptation. Third, cultural diversity and digital intensity create distinct but opposing boundary conditions: cultural differences attenuate leadership effectiveness by creating interpretive challenges and complicating the establishment of shared safety norms, while frequent digital tool usage surprisingly amplifies leadership effectiveness by providing multiple relationship-building touchpoints that compensate for the absence of face-to-face interaction. This pattern of findings challenges deterministic views that either celebrate or condemn virtual work arrangements, instead revealing that virtual team effectiveness depends critically on adaptive leadership that accounts for cultural complexity while leveraging rather than resisting technological mediation.

The theoretical contributions of this research are multifaceted and advance several streams of literature. First, the study extends leadership theory by demonstrating how traditional relationship-oriented behaviors manifest and operate effectively in digitally-mediated cross-cultural contexts, challenging assumptions that virtual environments inherently diminish interpersonal leadership effectiveness (Avolio and Gardner, 2005). Second, the research advances understanding of voice and silence as dynamic conversion processes rather than static behavioral states, providing a more nuanced theoretical framework for examining employee communication patterns. Third, the integration of social cognitive theory and leader-member exchange theory offers a comprehensive explanation for the psychological mechanisms through which leadership influences communication behaviors in complex organizational contexts.

The practical implications for cross-cultural virtual team management are substantial and actionable across multiple organizational levels. For leadership development programs, organizations should prioritize training that specifically addresses relationship-oriented behaviors in digital contexts, moving beyond traditional face-to-face leadership development. This includes explicit instruction on expressing consideration and support through text-based communication (using acknowledgment, appreciation, and empathetic language), leveraging video conferencing for relationship-building conversations (scheduling regular one-on-one check-ins beyond task discussions), and using digital collaboration platforms strategically to create informal interaction opportunities (virtual coffee chats, non-work channels, recognition posts). Training should include practice scenarios where leaders receive feedback on their digital communication style from diverse cultural perspectives, helping them recognize how their intended supportiveness might be interpreted differently across cultures.

For team composition and management, the cultural differences moderation effect suggests that organizations should implement differential support structures based on team diversity levels. Highly diverse teams require more intensive relationship-building investments from leaders, potentially including cultural liaisons or team facilitators who can bridge communication gaps, structured protocols for ensuring all cultural perspectives are heard, and extended team formation periods with explicit cultural norm discussions. Organizations might consider providing leaders of highly diverse teams with reduced team sizes or additional administrative support to allow sufficient time for individualized relationship building across cultural boundaries.

Regarding technology infrastructure, the positive moderating effect of digital tool usage indicates that organizations should invest not just in acquiring collaboration platforms but in promoting their frequent and strategic use for relationship purposes, not merely task coordination. This requires moving beyond viewing digital tools as productivity enablers to recognizing them as relationship-building media. Practical implementation includes establishing norms for regular synchronous video meetings (not just asynchronous updates), creating dedicated channels for social interaction and informal conversation, encouraging leaders to use multiple communication modalities to reach team members (mixing video, voice, instant messaging, and email based on message purpose and cultural preferences), and providing training on communication best practices for each platform type. Organizations should audit their digital tool portfolios to ensure leaders have access to platforms that support both task efficiency and relational depth.

For performance management systems, organizations should incorporate relationship-oriented competencies into leadership evaluation frameworks for virtual team leaders, with specific behavioral indicators adapted for digital contexts such as response timeliness to team member communications, frequency of individualized check-ins, demonstrated cultural sensitivity in digital interactions, and effectiveness in creating psychologically safe virtual environments. Multi-rater feedback from culturally diverse team members can assess whether leaders’ intended relationship-oriented behaviors are received as supportive across different cultural perspectives.

Finally, for organizational policy and culture, creating structures that legitimize relationship-building time in virtual environments is essential. This includes protecting calendar time for non-task-focused team interactions, recognizing relationship-building activities in workload allocations, and celebrating leaders who effectively build cohesive cross-cultural virtual teams. Organizations should communicate explicitly that relationship development is not a distraction from productivity but a prerequisite for it in virtual cross-cultural contexts, countering potential perceptions that visible task work should dominate virtual team interactions.

Several limitations constrain the interpretation and generalizability of these findings. First, the cross-sectional design limits causal inferences, despite the temporal separation of data collection phases implemented to reduce common method bias. While we collected data at three time points separated by two-week intervals, this approach does not fully capture the longitudinal dynamics of relationship development or the temporal sequences through which psychological safety mediates leadership effects on voice-silence conversion. Future longitudinal research should examine how relationship-oriented behaviors and voice-silence conversion patterns evolve over extended periods in virtual team contexts, ideally using diary studies or experience sampling methods to capture within-person variation in voice and silence behaviors across different situational contexts.

Second, while we addressed the nested data structure through ICC reporting and sensitivity analyses, our primary analysis treated individuals as independent observations due to sample size constraints at the team level. With 68 teams and an average of 5 team members per team, multilevel structural equation modeling would have limited statistical power to detect cross-level interactions or adequately partition within-team and between-team variance. Future research with larger numbers of teams could more rigorously model the multilevel nature of these phenomena, examining how team-level leadership climate and collective psychological safety interact with individual-level perceptions to influence voice-silence conversion.

Third, the conceptualization and measurement of voice-silence conversion captures employees’ general orientation toward behavioral flexibility and situational responsiveness rather than tracking actual moment-to-moment transitions between voice and silence states. While this approach provides valuable insights into dynamic communication tendencies, it does not capture the triggering events, contextual cues, or decision-making processes that prompt specific shifts from silence to voice or vice versa in real time. Future research employing intensive longitudinal designs such as event-triggered experience sampling could more directly observe and analyze the conversion process as it unfolds.

Fourth, the sample, while culturally diverse across 23 countries, represents primarily knowledge-intensive industries including technology, consulting, and finance sectors. The findings may not generalize to virtual teams in manufacturing, healthcare, or public sector organizations where work processes, hierarchical structures, and communication norms may differ substantially. Additionally, all participating organizations were large multinational corporations with established virtual team practices, potentially limiting generalizability to small and medium enterprises or organizations in early stages of virtual team adoption.

Fifth, despite procedural and statistical remedies, the reliance on self-reported survey data creates potential for social desirability bias, particularly for relationship-oriented behavior reports from leaders and voice behavior reports from employees. While temporal separation and anonymity assurances help mitigate these concerns, future research could triangulate self-reports with objective behavioral indicators such as communication frequency logs from digital platforms, supervisor ratings of employee voice, or peer assessments of leadership behaviors.

Future research directions should explore several critical areas to advance understanding of leadership and communication in cross-cultural virtual teams. First, longitudinal research designs are needed to examine the temporal dynamics of relationship development in virtual environments, particularly investigating how cultural differences and digital communication competencies influence the trajectory and pace of trust building over time (Zaccaro and Bader, 2003). Experience sampling methodology could capture daily fluctuations in psychological safety and voice-silence behaviors, revealing how discrete leadership actions or team events trigger shifts in communication patterns and identifying critical periods or thresholds in relationship development.

Second, investigating the role of specific digital technologies and platform features represents a promising avenue. Different collaboration tools—synchronous versus asynchronous communication platforms, video conferencing versus text-based channels, open forums versus private messaging—may differentially enable or constrain relationship-oriented leadership expression and psychological safety development. Experimental or quasi-experimental designs could systematically vary digital communication modalities to identify which technological features best support relationship-oriented leadership in culturally diverse contexts.

Third, research should examine potential curvilinear relationships or interaction effects among moderating variables. For instance, does very high digital tool usage frequency eventually diminish leadership effectiveness through information overload or reduced personal connection? Do certain combinations of cultural diversity dimensions (e.g., high power distance combined with high collectivism) create unique challenges or opportunities for relationship-oriented leadership that linear models cannot capture?

Fourth, expanding the nomological network to include additional mediating mechanisms would provide a more complete theoretical account. Potential additional mediators include trust, team cohesion, communication quality, and shared mental models, which may operate in parallel or sequence with psychological safety to transmit leadership effects on voice behaviors. Multi-mediator models could clarify which psychological pathways are most critical for different types of voice (promotive versus prohibitive) or different cultural contexts.

Fifth, investigating boundary conditions related to leader characteristics would enhance practical applicability. Do leaders’ cultural intelligence, digital communication competence, or emotional intelligence moderate their ability to effectively deploy relationship-oriented behaviors in virtual cross-cultural settings? Identifying the competencies that enable some leaders to overcome cultural and technological barriers more effectively than others would inform selection and development practices.

Finally, comparative research across different types of virtual teams—global virtual teams versus domestic culturally diverse teams, permanent teams versus temporary project teams, fully virtual versus hybrid configurations—could identify which findings generalize broadly versus which are specific to particular virtual team contexts. Such comparative work would build a more nuanced, contextually sensitive theory of leadership and communication in digitally-mediated organizational environments.

For management practice, organizations should develop comprehensive training programs that enhance leaders’ abilities to build relationships and foster psychological safety in virtual environments while accounting for cultural diversity. The findings emphasize the critical importance of relationship-oriented leadership as a strategic capability for managing cross-cultural virtual teams, with implications for leadership selection, development, and performance evaluation systems in increasingly digital and globalized organizational contexts.

## Data Availability

The original contributions presented in the study are included in the article/supplementary material, further inquiries can be directed to the corresponding author.
